# Establishment of dry chemistry based reference intervals of renal function test parameters for the adult population of Kaski District, Nepal

**DOI:** 10.1186/s12882-021-02542-4

**Published:** 2021-10-06

**Authors:** Goma Kathayat, Daya Ram Pokharel, Naval Kishor Yadav, Manoj Sigdel

**Affiliations:** Department of Biochemistry, Manipal College of Medical Sciences and Teaching Hospital, Pokhara, Nepal

**Keywords:** Reference interval, Dry chemistry, Sodium, Potassium, Urea, Creatinine, Kaski, Nepal

## Abstract

**Background:**

Reference intervals (RIs) for clinical chemistry test parameters are specific to the method of measurement and population under service. However, there has been no locally available dry chemistry based RIs for the Nepalese population. Thus, the present study aimed to establish dry chemistry based RIs for sodium, potassium, urea, and creatinine specific to adult populations of Kaski districts, Nepal

**Methods:**

This was a cross-sectional study conducted at the Manipal Teaching Hospital, Pokhara, Kaski, Nepal on 360 healthy adult participants aged 18–65 years. The test parameters under study were analyzed using a fully automated OCD Vitros 350 dry chemistry analyzer following the protocols provided by the reagent kit manufacturer. The RIs were estimated using reference limits at 2.5th and 97.5th percentiles. The normal distribution of the data was tested by Kolmogorov–Smirnov, and Shapiro–Wilk tests. The differences between males and females RIs were compared by the Mann-Whitney test while age-specific RIs for each sex was compared by One-Way-ANOVA and Dunnett’s Multiple Comparisons Tests. All the data were managed and analyzed using MS Excel and SPSS version 20.

**Results:**

The RIs of urea, creatinine, sodium, and potassium specific to the adult population of Kaski district, Nepal are as follows: urea: 4.20–13.70 mmol/L (males: 4.70–13.99; females: 4.20–13.23); creatinine: 44.20–106.10 μmol/L (males: 48.82–106.10; females: 35.40–83.78); sodium 135–146 mmol/L (males: 135–146; females: 135–146) and potassium 3.60–5.10 mmol/L (males: 3.54–5.0; females: 3.60–5.10). These RIs were found to be different from currently used RIs provided by the reagent manufacturer. RIs of all the test parameters were significantly influenced by the age of the study participants. However, only the RIs of urea, creatinine, and potassium were significantly influenced by sex.

**Conclusions:**

The present study has for the first time established dry chemistry based RIs for selected renal function test parameters specific to the adult population of Kaski district, Nepal. This result will aid the clinician in minimizing the errors in result interpretation and making a precise clinical decision.

## Background

Reference intervals (RIs) refer to the quantitative data of clinical chemistry parameters accompanied by upper and lower limits [1]. They serve as the basis for laboratory testing and assist physicians in distinguishing between healthy and diseased patients. They are also used to interpret the results of laboratory measurements, the screening of clinical trials, and as a basis for safety monitoring for trial participants [2]. Population-specific RIs for quantitative clinical chemistry parameters are established according to the published guidelines of the clinical and Laboratory Standards Institute (CLSI 2008) [3] and the International Federation for Clinical Chemistry and Committee on Reference Intervals and Decision Limits (IFCC-CRIDL) [4]. CLSI and IFCC publish and updates these guidelines regularly for manufacturers and laboratories to accomplish their own RI studies. This is because measured values of clinical laboratory parameters are not only influenced by individual factors such as age, sex, and lifestyle, but also by the method of estimation, race, and ecological factors like climate and altitude. Besides, they also vary not only between individuals but also between populations [1, 5]. Globally, the RIs in use is usually referred from the textbooks or research articles or values provided in the reagent kit insert. Thus, it is not suitable to use the RIs that do not symbolize the local population and the method being used. So, for the interpretation of individual patient laboratory test results, the testing method and population based RIs are the most widely used tools [2]

In Nepal, the majority of the diagnostic laboratories provide liquid chemistry based testing services. The RIs for such testing are widely adopted from the scientific literature, clinical chemistry textbooks, and commercially provided kit inserts. Since 2018 there has been a gradual introduction of dry chemistry based testing platforms as well in selected laboratories and hospitals of Nepal. However, the RIs for such dry chemistry based testing platforms are rarely available in clinical chemistry textbooks and published scientific literature as compared to the liquid-based chemistry parameters. The only source of these values is kit inserts provided by the reagent manufacturers which are not specific to the local population being served. Hence, in order to address this gap, there is an urgent need for establishing RIs that are both method and population specific. To the best of our knowledge, there has been the establishment of liquid chemistry based RIs only for lipid profile parameters for the Nepalese population [6]. There has been not a single scientific report on dry chemistry based RIs for the whole population of Nepal. Therefore, the present study attempts to fill up this twofold research gap by establishing dry chemistry based RIs for selected renal function test parameters among adult populations of Kaski district, Nepal.

## Methods

### Study design

A laboratory-based cross-sectional study was conducted at the Department of Clinical Biochemistry of Manipal Teaching Hospital, Kaski, Nepal for a period of 6 months from June 2020 to November 2020 following the guidelines provided by CLSI and IFCC-CRIDL [3, 4].

### Study population and selection criteria

A total of 360 healthy adult participants of Kaski district, Nepal aged between 18 and 65 years were selected and enrolled using the priori convenient sampling technique. These adult participants were either the patient’s attendees or students and staffs of Manipal Teaching Hospital, Pokhara, Nepal. Study participants with one or more of the following conditions were excluded from the study:

- hypertension, kidney failure, cardiac diseases, chronic respiratory diseases, liver diseases, malabsorption syndromes, diabetes mellitus, malignancies, and hematological disorders including anemias
- history of being a hospital in-patient or otherwise seriously ill during the previous 4 weeks
- known carrier state for hepatitis virus B (HBV), hepatitis virus C (HCV), or Human immunodeficiency virus (HIV)
- undergoing or has recently undergone a replacement or supplementation therapy e.g., thyroxine, insulin
- having psychological and mental disorders
- female participants who are pregnant, breastfeeding, or within 1 year of childbirth
- BMI ≥30 kg/m^2^

### Anthropometric, physiological, and lifestyle-related variables

All the study participants were interviewed within the hospital premises using a pre-validated set of questionnaires and information regarding age, sex, disease if any, family history, race, dietary habits, physical activity, smoking, or alcohol intake habits were recorded. Participants who reported drinking ethyl alcohol on a regular or occasional basis were labeled as alcoholics while those who never had alcohol in their life or quit it for the last 1 year were labeled as non-alcoholics. Body height, weight, waist circumference (WC), hip circumference (HC), waist-hip ratio (WHR), and body mass index (BMI) of the participants were measured following the standard protocols [7]. According to the WHO guidelines for the South Asian population BMI of < 18.5 was classified as underweight; 18.5–22.9 as normal weight; 23–24.9 as overweight; 25–29.9 as Obese I and ≥ 30 as Obese II [8]. Blood pressure was measured using a digital sphygmomanometer (Accumed automatic upper arm blood pressure monitor, Swiss design) in triplicate while in the sitting position after about five minutes of rest.

### Sample collection, processing, and storage

About 5 ml of venous blood samples were collected from each participant in gel tubes with clot activators between 6 to 9 a.m. after they underwent a minimum of 8–10 h fasting. The collected blood samples were allowed to clot for about 20–30 min at room temperature and then centrifuged at 4000 rpm for 10 min. Samples that were lipemic, hemolytic, or icteric were rejected and not included in the study. The serum samples so obtained were analyzed immediately whenever possible or stored at -20^o^ C until analyzed in case of delay.

### Biochemical analysis

The concentrations of serum urea, creatinine, sodium, and potassium were measured using a fully automated dry chemistry based analyzer (VITROS® 350 chemistry system, Ortho clinical diagnostics, UK) according to the standardized protocols provided by the manufacturers. Briefly, serum urea was measured by the urease method, creatinine was measured by a two-point rate enzymatic method which involves the use of creatinine amidohydrolase, sarcosine oxidase, and peroxidase and leuco dye. This method was validated and the values were traceable to a Gas Chromatography Isotope Dilution Mass Spectrometry (GC/IDMS) method and National Institute of Standards and Technology (NIST) SRM® 914 creatinine standard reference material. Sodium and potassium were measured using the ion-selective electrode (ISE) method based on the principles of direct potentiometry. All specimens from each individual were assayed in a single batch, using the same lots of reagents to minimize the analytical variation.

### Quality control and quality assurance

Analysis of samples was done after proper standardization of the instruments with the help of calibrators and internal controls. We ran two levels of quality control (QC) sera for each analyte under the study and calculated both inter-and intra-assay coefficients of variation (CV%). Inter-assay CV% (reproducibility) for serum urea, creatinine, sodium, and potassium were 2.12, 2.62, 1.08, and 2.75, respectively. Similarly, intra-assay CV% (repeatability) for urea, creatinine, sodium, and potassium were 0.96, 1.10, 1.08, and 0.39, respectively. All of these CV% were within the acceptable ranges. Internal QC was performed every day and observed values were within ±2SD from their target values. Our laboratory also takes part in the monthly External Quality Assessments Scheme (EQAS) run by the Christian Medical College* (*CMC) Vellore, India to guarantee the accuracy and reliability of our laboratory test values.

### Statistical analysis

All data management and statistical analyses were performed using MS Excel, Statistical Package for the Social Sciences (SPSS) version 20 (SPSS, IBM, Chicago, IL), and GraphPad Prism version 9. The data were categorized based on the sex and age groups of the study participants. The outliers present in the data were recognized with the help of Box plots, a procedure recommended by Horn and Pesce [9]. They were manually deleted which led to different sample sizes for each parameter.

The normal distribution of the study data was tested by D’Agostino-Pearson Omnibus, Kolmogorov–Smirnov, and Shapiro–Wilk tests. Our data were found to follow the non-Gaussian probability curve. Therefore, nonparametric statistical methods were used to establish the RIs as per the CLSI C28-A3 guideline [3]. RIs were calculated as the 2.5 percentile confidence interval (P 2.5) and 97.5 percentile confidence interval (P 97.5) separately for both males and females. The Mann-Whitney test was used to compare the RIs between males and females while One-Way-ANOVA and Dunnett’s multiple comparisons tests were used for the comparison of age group-specific RIs for each sex. All *p* values (two-tailed) < 0.05 were considered statistically significant.

## Results

The serum levels of urea, creatinine, sodium, and potassium displayed non-Gaussian distribution. Table 1 shows the sociodemographic and anthropometric variables of study participants according to their sex. Of the 360 healthy participants studied, 180 (50%) were males and 180 (50%) were females. The adult age ranged from 18 to 65 years and the mean age of the participants was 40.38 ± 14.55 years. The age group intervals of the participants were categorized as 18–29 years; 30–39 years; 40–49 years and 50–65 years. There were 99 (27.5%) participants within age group 18–29 years, 73 (20.3%) in 30–39 years, 76 (21.1%) in 40–49 years and 112 (31.1%) in 50–65 years. All the participants were normotensive with the mean systolic blood pressure (SBP) of 116.61 ± 10.36 mmHg and diastolic blood pressure (DBP) of 77.11 ± 8.51 mmHg. The mean height of the participants was 158.88 ± 9.17 cm. Overall, the mean BMI of the participants was 23.82 ± 2.28 kg/m^2^. Based on WHO guidelines for South Asian population, there were 113 (31.4%) participants classified as normal weight BMI, 112 (31.1%) as overweight, and 135 (37.5%) as obese I. The mean WC of the participants was 89.69 ± 11.40 cm and WHR was 0.96 ± 0.12. Based on their racial background, 245 (68.1%) were Aryans, 61 (16.9%) were Mongolians, 35 (9.7%) were Dalits and 19 (5.3%) were Newars. Among all the participants, 314 (87.2%) were non-vegetarians and 46 (12.8%) were vegetarians. The majority of the participants were non-smokers (334, 92.8%) and non-alcoholics (212, 58.9%). Likewise, the mean values of serum urea, creatinine, height creatinine ratio, sodium and potassium were 8.4 ± 2.5, 66.5 ± 17.1, 224.2 ± 55.0, 141.2 ± 2.8, and 4.3 ± 0.4 respectively. There were no significant sex differences (*p* > 0.05) in age, age groups, SBP, DBP, BMI, race, serum sodium and potassium. However, significant sex differences were observed in terms of height, WC, WHR, diet, alcohol intake, smoking, serum urea, creatinine and height creatinine ratio (*p* < 0.05).

**Table 1:** Sociodemographic and anthropometric variables of study participants

Characteristic Variables	Male	Female	***p***-value	Total
**N (%)**	180 (50)	180 (50)		360 (100)
**Age (years)**	40.86 ± 14.68	39.90 ± 14.43	0.534^c^	40.38 ± 14.55
**Age groups (years)**				
18–29	47 (26.1)	52 (28.9)	0.708^c^	99 (27.5)
30–39	39 (21.7)	34 (18.9)		73 (20.3)
40–49	35 (19.4)	41 (22.8)		76 (21.1)
50–65	59 (32.8)	53 (29.4)		112 (31.1)
**Blood pressure (mmHg)**				
Systolic	117.53 ± 10.10	115.69 ± 10.57	0.091^c^	116.61 ± 10.36
Diastolic	77.46 ± 8.53	76.76 ± 8.51	0.436^c^	77.11 ± 8.51
**Height (cm)**	163.27 ± 8.48	154.49 ± 7.60	< 0.001^a^	158.88 ± 9.17
**BMI (kg/m**^**2**^**)**	23.92 ± 2.67	23.71 ± 2.29	0.374^c^	23.82 ± 2.28
**BMI (kg/m**^**2**^**)**				
Normal weight	54 (30)	59 (32.8)	0.446^c^	113 (31.4)
Over weight	55 (30.6)	57 (31.7)		112 (31.1)
Obese I	71 (39.4)	64 (35.6)		135 (37.5)
**Waist (cm)**	92.56 ± 10.26	86.82 ± 11.78	< 0.001^a^	89.69 ± 11.40
**W/H ratio**	1.02 ± 0.12	0.90 ± 0.09	< 0.001^a^	0.96 ± 0.12
**Race**				
Aryan	125 (69.4)	120 (66.7)	0.749^c^	245 (68.1)
Mongolian	27 (15)	34 (18.9)		61 (16.9)
Dalit	19 (10.6)	16 (8.9)		35 (9.7)
Newar	9 (5.0)	10 (5.6)		19 (5.3)
**Diet**				
Vegetarian	15 (8.3)	31 (17.2)	0.012^b^	46 (12.8)
Non-vegetarian	165 (91.7)	149 (82.8)		314 (87.2)
**Alcohol Intake**				
No	60 (33.3)	152 (84.4)	< 0.001^a^	212 (58.9)
Yes	120 (66.7)	28 (15.6)		148 (41.1)
**Smoking**				
No	155 (86.1)	179 (99.4)	< 0.001^a^	334 (92.8)
Yes	25 (13.9)	1 (0.6)		26 (7.2)
**Serum urea (mmol/L)**	8.9 ± 2.4	7.9 ± 2.4	< 0.001^a^	8.4 ± 2.5
**Serum creatinine (μmol/L)**	76.7 ± 15.4	56.4 ± 12.1	< 0.001^a^	66.5 ± 17.1
**Height-creatinine ratio**	196.2 ± 43.2	252.3 ± 51.2	< 0.001^a^	224.2 ± 55.0
**Serum sodium (mmol/L)**	141.1 ± 2.9	141.2 ± 2.8	0.827^c^	141.2 ± 2.8
**Serum potassium (mmol/L)**	4.4 ± 0.4	4.3 ± 0.4	0.114^c^	4.3 ± 0.4

The results are presented as mean ± SD for continuous variables and n (%) for categorical variables ^a^*p* < 0.001, ^b^*p* < 0.05, ^c^*p* > 0.05 (two tailed); groups were compared using Students t test for quantitative variables and Chi square test for categorical variables. For the conversion of SI unit to conventional unit divide the concentration of urea value by 0.357, creatinine value by 88.4 and sodium and potassium value by 1

Table 2 shows the total and sex-specific RIs for renal function test parameters under study*.* The RI for urea: 4.20–13.70 mmol/L (males: 4.70–13.99 mmol/L; females: 4.20–13.23 mmol/L; *p*-value = < 0.001); creatinine: 44.20–106.10 μmol/L (males: 48.82–106.10 μmol/L; females: 35.40–83.78 μmol/L; *p*-value = < 0.001); sodium 135–146 mmol/L (males: 135–146; females: 135–146; *p*-value = 0.831) and potassium 3.60–5.10 mmol/L (males: 3.54–5.0; females: 3.60–5.10; *p*-value = 0.032). Urea, creatinine, and potassium RIs showed significant sex differences (*p* < 0.05) while the RI for sodium showed no significant sex difference (*p* > 0.05).

**Table 2:** The established reference intervals for renal function test parameters for male and female adults

Analytes	Sex	Unit	N	Median	Percentiles		Reference Values	Interval values	Difference between Male and Female	
					2.5	97.5			z-value	***p***-value
**Urea**	Male	mmol/L	178	8.70	4.70	13.99	4.70–13.99	9.29	−3.966	< 0.001
		mg/dL		24.25	13.09	38.40	13.09–38.40	25.31		
	Female	mmol/L	178	7.50	4.20	13.23	4.20–13.23	9.03		
		mg/dL		20.85	11.80	36.20	11.80–36.20	24.40		
	Total	mmol/L	356	8.05	4.20	13.70	4.20–13.70	9.50		
		mg/dL		22.20	11.89	37.81	11.89–37.81	25.92		
**Creatinine**	Male	μmol/L	180	79.60	48.82	106.10	48.82–106.10	57.28	−11.983	< 0.001
		mg/dL		0.90	0.55	1.20	0.55–1.20	0.65		
	Female	μmol/L	180	53.00	35.40	83.78	35.40–83.78	48.38		
		mg/dL		0.60	0.40	0.90	0.40–0.90	0.5		
	Total	μmol/L	360	61.90	44.20	106.10	44.20–106.10	61.9		
		mg/dL		0.70	0.50	1.20	0.50–1.20	0.7		
**Sodium**	Male	mmol/L/	178	141	135	146	135–146	11		
	Female	mEq/L	180	141	135	146	135–146	11	−0.214	0.831
	Total		358	141	135	146	135–146	11		
**Potassium**	Male	mmol/L/	176	4.40	3.54	5.00	3.54–5.00	1.5		
	Female	mEq/L	177	4.30	3.60	5.10	3.60–5.10	1.5	−2.145	0.032
	Total		353	4.30	3.60	5.10	3.60–5.10	1.5		

The results are presented as both medians and the percentiles. The RIs are presented as the values between 2.5 and 97.5 of the percentile. The number of participants is indicated under the column labeled N. The RIs with *p*-values (two tailed) < 0.05 are the ones that differ significantly between male and female

Table 3 compares the RIs based on the age groups of the study participants. Since all age groups did not have a minimum sample size of 120 as required by CLSI and IFCC-CRIDL guidelines, the comparison of age-specific RIs was carried out by using One-Way-ANOVA and Dunnett’s Multiple Comparisons Test. The mean RIs of all four test parameters were significantly different in each age group (*p* < 0.05). The mean RI was significantly different between age group 18–29 years and age group 30–39 years for sodium; age group 18–29 years and age group 40–49 years for sodium and potassium and age group 18–29 years and age group 50–65 years for urea, creatinine, and potassium.

**Table 3:** Comparison of reference intervals for male and female in the different age group for renal function test parameters

Analytes	Sex	Unit	N	18–29 years	N	30–39 years	N	40–49 years	N	50–65 years	***p***-value
**Urea**	Male	mmol/L	47	8.52 ± 2.30*	39	8.42 ± 2.28	35	8.60 ± 2.17	57	9.54 ± 2.63	0.240
		mg/dL		23.88 ± 6.43*		23.60 ± 6.37		24.09 ± 6.07		25.96 ± 6.72	
	Female	mmol/L	52	6.81 ± 2.01	34	7.51 ± 2.09	41	7.90 ± 2.10^b^	51	9.08 ± 2.74^c^	< 0.001
		mg/dL		19.08 ± 5.63		21.03 ± 5.82		22.15 ± 5.89^b^		24.80 ± 6.60^**c**^	
	Total	mmol/L	99	7.62 ± 2.30	73	8.11 ± 2.22	76	8.22 ± 2.15	108	9.32 ± 2.68^c^	< 0.001
		mg/dL		21.36 ± 6.46		22.40 ± 6.22		23.01 ± 6.01		25.41 ± 6.66^**c**^	
**Creatinine**	Male	μmol/L	47	72.03 ± 16.10*	39	77.29 ± 13.86*	35	78.04 ± 14.30*	59	79.11 ± 15.95*^c^	0.048
		mg/dL		0.82 ± 0.18*		0.87 ± 0.16*		0.88 ± 0.16*		0.91 ± 0.18*^c^	
	Female	μmol/L	52	52.86 ± 12.80	34	55.62 ± 9.86	41	56.70 ± 10.64	53	60.21 ± 12.99^c^	0.060
		mg/dL		0.60 ± 0.14		0.63 ± 0.11		0.64 ± 0.12		0.67 ± 0.13^**c**^	
	Total	μmol/L	99	61.96 ± 17.30	73	67.20 ± 16.26	76	66.53 ± 16.37	112	70.16 ± 17.37^c^	0.006
		mg/dL		0.70 ± 0.20		0.76 ± 0.18		0.75 ± 0.19		0.79 ± 0.20^**c**^	
**Sodium**	Male	mmol/L/	47	139.85 ± 3.10	39	141.79 ± 2.49^a^	34	142.15 ± 2.46^b^	58	141.14 ± 2.85	0.001
	Female	mEq/L	52	140.52 ± 3.10	34	141.85 ± 2.16	41	141.93 ± 2.30^b^	53	140.89 ± 2.89	0.033
	Total		99	140.20 ± 3.10	73	141.82 ± 2.33^**a**^	75	142.03 ± 2.36^**b**^	111	141.02 ± 2.86	< 0.001
**Potassium**	Male	mmol/L/	47	4.26 ± 0.36	39	4.35 ± 0.32	34	4.41 ± 0.32	56	4.41 ± 0.39	0.138
	Female	mEq/L	51	4.22 ± 0.38	34	4.31 ± 0.26	39	4.35 ± 0.29	53	4.32 ± 0.42	0.321
	Total		98	4.24 ± 0.37	73	4.33 ± 0.29	73	4.38 ± 0.30^**b**^	109	4.36 ± 0.41^**c**^	0.033

RIs are expressed as mean ± standard deviation while the number of participants is shown in column labeled N. *indicates the differences of RIs between males and females of the same age group are statistically significant (*p* < 0.05 by student’s t test)

^a^ represents the significant difference of each analyte between age group 18–29 and 30–39 years for same-sex where *p* < 0.05 by One-Way ANOVA and Dunnett’s Multiple Comparison Test

^b^ represents the significant difference of each analyte between age group 18–29 and 40–49 years for same-sex where *p* < 0.05 by One-Way ANOVA and Dunnett’s Multiple Comparison Test

^c^ represents the significant difference of each analyte between age group 18–29 and 50–65 years for same-sex where *p* < 0.05 by One-Way ANOVA and Dunnett’s Multiple Comparison Test

A comparison of our estimated RIs for urea, creatinine, sodium, and potassium with the ones provided by the manufacturer and currently being used at our laboratory is shown in Table 4**.**

**Table 4:** Comparison of reference interval of this study with adopted reference interval

Analytes	Sex	Unit	Reference interval established in the present study	Adopted reference interval provided for the dry chemistry based platforms (OCD Vitros)
**Urea**	Male	mmol/L	4.70–13.99	3.2–7.1
		mg/dL	13.09–38.40	9–20
	Female	mmol/L	4.20–13.23	2.5–6.1
		mg/dL	11.80–36.20	7–17
	Total	mmol/L	4.20–13.70	–
		mg/dL	11.89–37.81	–
**Creatinine**	Male	μmol/L	48.82–106.10	58–110
		mg/dL	0.55–1.20	0.66–1.25
	Female	μmol/L	35.40–83.78	46–92
		mg/dL	0.40–0.90	0.52–1.04
	Total	μmol/L	44.20–106.10	–
		mg/dL	0.50–1.20	–
**Sodium**	Male	mmol/L/	135–146	–
	Female	mEq/L	135–146	–
	Total		135–146	137–145
**Potassium**	Male	mmol/L/	3.54–5.00	–
	Female	mEq/L	3.60–5.10	–
	Total		3.60–5.10	3.50–5.10

## Discussion

There has been a complete lack of dry chemistry based RIs of biochemical test parameters for the entire age groups of the Nepalese population. The present study aimed to establish the RIs of serum urea, creatinine, sodium, and potassium for the adult population of the Kaski district, Nepal. Due to the lack of locally established RIs, Nepalese clinical laboratories tend to adopt RIs established for foreign populations directly from the reagent kit inserts or some clinical chemistry textbooks without further validation. The reason for going into such practices is possibly due to the cost factors, lack of awareness, and no mandatory instructions from the local regulatory bodies. The role of clinical laboratories is not only to generate numerical test results but also to guide clinicians to make the better screening, diagnosis, prognosis, and treatment of the diseases, and to conduct successfully any planned clinical trials. However, these roles are genuinely fulfilled only when the test results are interpreted with reference to RIs specific to the population being served or studied. In their absence, there is always a chance of wrong interpretation of the test results, which may lead to the wrong diagnosis and management of the target diseases. Besides, the wrong diagnosis and treatment may affect the local population in terms of their financial resources, health status, and time. This is the reason why international regulatory bodies such as CLSI and IFCC recommend that each clinical laboratory should establish its RIs [3, 4]. In addition to the generalized RIs, the establishment of RIs with respect to sex, age group, pathophysiological status, and race of the general population provides extra resolution for better test results interpretation and clinical decision making. We thus also aimed to establish the sex-based RIs for the test parameters under study. Though we have reported the age group-specific RIs, their validity remains questionable as the sample size for each age group was less than that recommended by the CLSI and IFCC guidelines.

Dry chemistry based platform was introduced in the diagnostic market very recently hence there are very few literatures available on the establishment of RIs based on these new platforms. Thus, we compare our study findings with the previously established RIs that were mostly based on liquid chemistry based platforms.

The RIs for serum urea and creatinine were significantly higher in males (4.70–13.99 mmol/L and 48.82–106.10 mmol/L) as compared to females (4.20–13.23 mmol/L, and 35.40–83.78 mmol/L). This sex-based difference may be associated with the previously reported factors such as higher muscle masses and height in males compared to females, including others such as differences in nutrition, and physiological status. Similar findings have been reported in populations of North India, China, Tanzania, Uganda, Kericho; Kenya, and North-Rift Valley; Kenya [2, 10–14]. On the contrary, there was no significant sex difference for urea and creatinine in a study conducted in the Nigerian population [15]. There was no significant difference in RI for sodium between males and females**.** This finding contradicts the findings of a study conducted in China, North-Rift Valley; Kenya, and Rwanda where a significant difference in RI for sodium was observed between males and females [10, 14, 16]. There was a significant sex difference for potassium with a high RI of potassium present among the female participants (Table 2). This could be explained by the fact that females have higher platelet counts than males [17] that leads to additional serum potassium that results from platelet rupture during coagulation [18, 19]. On the other hand, males have significantly higher RI for potassium than females in a population of North-Rift Valley, Kenya [14].

We also attempted to analyze age group and sex-specific RIs in our study samples. Overall, we found that RIs for all four test parameters were significantly different for each age group. The observed differences in RIs suggest that the serum concentration of these parameters is age-dependent (Table 3). The age-specific differences in the serum values of urea and creatinine could be explained by the fact that with the advancing age there is an increase in muscle degradation and a decrease in glomerular filtration rate in both sexes. Comparable findings have been observed in the population of North India, and North-Rift Valley; Kenya [2, 14]. The sex-wise analysis further revealed that only the RIs of serum urea for females, creatinine for males, and sodium for both males and females differed significantly among different age groups. There was an increase in the mean values of urea with the increase in age among female participants. A similar study done in the Rwandan population found that serum urea levels in females showed a slight decline with age [16]. Similarly, there was an increase in the mean values of creatinine with the increase in age among male participants. Conversely, there was an increase in serum creatinine with an increase in age for females in a study done in North-Rift Valley; Kenya [14]. These inconsistencies may be due to demographic, geographic, and racial differences. Besides, the hidden pathologies, the variability of analytical methods, types, and analytical performance of the equipment and the quality of the reagents being used might have influenced the value of these parameters in these studies.

The combined effect of genetic and environmental factors, variable nature of physical activities, and age-related decline in renal function could be attributed to the observed differences in the mean values of serum sodium and potassium with the increase in age. Besides, serum potassium intake depends on habits, race, diet, and so on. Survey data from the Third National Health and Nutrition Examination Survey (NHANES III, United States) demonstrated that irrespective of the sex of the study subjects, the mean potassium consumption of the American people increased with age upto the age group of 31–50 years. Thereafter, following the age of 50 years, the daily K^+^ intake slowly and noticeably decreases [20]. However, no clinically significant differences were observed in different age-specific RIs for potassium in the study done in China [21].

The dry chemistry based RIs that are provided in the kit inserts and currently being used at our laboratory reporting were found to be different than what we established for our local population of the Kaski district of Nepal. Not only these but our RIs were also found to differ to some extent with the RIs determined elsewhere by the same methodology and analytical platforms [2, 5, 10, 14]. These observed differences could be explained in the light of sample size, genetic, race, demographic, geographical location, diet, lifestyle, cultural and seasonal differences among the studied populations [1, 2, 5, 22]. This is the reason why several studies [5, 10, 23] and international guidelines [3, 4] recommend every diagnostic laboratory establish their RIs specific to the local population being served and not blindly follow the RIs for unrelated populations.

### Strengths and limitations

This was the first study from Nepal designed as per the IFCC guidelines to establish the dry chemistry based RIs for the adult population of Kaski district, Nepal. The renal function test parameters were analyzed with well-established laboratory methods using a fully automated dry chemistry platform under strict internal and external quality assurance programs. Hence, the results generated through this study are accurate, precise, and reliable. The study participants included in this study represented different localities, socioeconomic strata, and major racial groups of Kaski District, Nepal.

This study was limited to the establishment of RIs only for the renal function test parameters of the adult population of the Kaski district. Thus, the RIs for the pediatric and geriatric age groups of this population remain to be established. It was also not possible to establish the valid adult RIs based on the age groups, race, and dietary habits of our study population due to the relatively small sample size, lack of standardized data on dietary habits, and limited resources. Thus, our RIs are best applicable only to the local adult population of the Kaski district and for dry chemistry based testing platforms. They may not apply to the similar age group populations of other regions of Nepal without further validation.

## Conclusions

This study establishes generalized and sex-specific dry chemistry based RIs for selected renal function test parameters to be used in the adult population of Kaski district, Nepal. It also opens a possibility for similar studies to be carried out in other parts of Nepal for various other age groups and clinical chemistry test parameters. This will ensure better assessment and interpretation of laboratory test results, thus improving the quality of healthcare in this region. Our RIs could also be adopted by the clinical laboratories of neighboring districts after their local validation. We recommend clinicians and researchers use these RIs for the population of Kaski district, Nepal with confidence while interpreting and analyzing their laboratory results. Our study also opens the avenue for the conduction of similar studies in other regions of the country to fill up the gap of RIs for the Nepalese populations.

## Data Availability

The datasets generated and analyzed during the current study would be available from the corresponding author on personal request.

## Abbreviations

BMI: Body mass index
CLSI: Clinical and Laboratory Standards Institute
CV: Coefficient of variation
°C: Degree celsius
DBP: Diastolic blood pressure
EQAS: External quality assessments scheme
HBV: Hepatitis B virus
HCV: Hepatitis C virus
HIV: Human immunodeficiency virus
IFCC-CRIDL: International Federation for Clinical Chemistry and Laboratory Medicine and Committee on Reference Intervals and Decision Limits
ISE: Ion-selective electrode
OCD: Ortho clinical diagnostics
QC: Quality control
RI: Reference interval
rpm: Rotation per minute
SBP: Systolic blood pressure
SD: Standard deviation
SPSS: Statistical Package for the Social Sciences
WC: Waist circumference
WHO: World Health Organization
WHR: Waist-hip ratio
